# Predicted Sensory Modality Determines the Timing and Topographies of Omitted Stimulus Potentials

**DOI:** 10.1111/psyp.70097

**Published:** 2025-06-25

**Authors:** Tomomi Ishida, Hiroshi Nittono

**Affiliations:** ^1^ Graduate School of Human Sciences The University of Osaka Osaka Japan

**Keywords:** event‐related potential, omission, prediction, predictive coding, self‐generation, sensory modality

## Abstract

It is thought that our brains actively predict what will happen next in the environment, but it remains unclear how specific the prediction of an upcoming event is. This study investigated whether the prediction about the sensory modality of the upcoming stimulus modulates neural responses to unexpected omissions of stimuli. Previous research has reported that the peak latencies of omitted stimulus potentials (OSPs) are shorter in the auditory modality than in the visual modality when tested in separate blocks. In this study, we presented auditory and visual stimuli in a fixed alternating pattern to examine whether modality‐specific OSPs occur even within a single block. Participants (*N* = 33) were asked to press a mouse button at a constant interval of 1 s. Each button press triggered either an auditory or visual stimulus, and these were presented twice in an alternating pattern (A, A, V, V, A, A, etc.). The stimuli were omitted in 12% of the trials. This method ensured each type of omission (of either auditory or visual stimuli) to be preceded equally often by either an auditory or a visual stimulus, thereby controlling for late event‐related potential components of the preceding stimulus, if any. The results showed that auditory OSPs had shorter peak latencies than visual OSPs and that their scalp topographies differed; auditory OSPs had more anterior and central distributions than visual OSPs. These findings suggest that OSPs occur in a modality‐specific manner according to the predicted sensory modality of the upcoming stimulus.

## Impact Statement

To investigate the specificity of the brain's sensory prediction process, neural responses to unexpected stimulus omissions were recorded in a task in which an auditory or visual stimulus was alternately presented twice by the participant's button press. Infrequently omitted stimuli elicited omitted stimulus potentials (OSPs) with different peak latency and scalp topography depending on the expected stimulus modality. The results suggest that predictions are modality‐specific, and such specific predictions are reflected in qualitatively different OSPs.

## 1 Introduction

In recent years, predictive coding theory has gained wide acceptance as a foundational framework for sensory information processing. According to this theory, the brain actively predicts upcoming sensory stimulation to efficiently interpret the external world. In this process, neural responses are continuously adjusted to minimize the discrepancy between predicted sensory input and actual sensory input—referred to as prediction errors (Arnal and Giraud 2012; Friston 2005; Friston and Kiebel 2009; Rao and Ballard 1999). The sensory activation pattern induced by the predicted input is believed to be represented as a neural template in lower‐level sensory areas (Mumford 1992). However, some questions remain to be discussed: To what extent are these predictions detailed? Are they specific to the physical characteristics of the stimulus or more general, reflecting a broad expectation that “something will happen”?

Omitted stimulus potentials (OSPs) are an empirical tool for investigating the mechanisms of sensory prediction, event‐related potentials (ERPs), that occur when an expected stimulus is unexpectedly omitted (for reviews, see Bendixen et al. 2012; Braga and Schönwiesner 2022; Heilbron and Chait 2018; Schröger et al. 2015). OSPs are considered markers of prediction error signals and their processing, reflecting “an exact mirror image” of the top‐down prediction (SanMiguel, Saupe, et al. 2013) in the absence of external sensory input. Most studies on omission responses have employed oddball paradigms in which stimulus omissions function as the deviant (e.g., Bullock et al. 1994; Cornella et al. 2015; Czigler et al. 2006; Hernández and Hernández‐Sánchez 2017; Horváth et al. 2010; Oceák et al. 2006; Prete et al. 2022; Salisbury 2012; Simson et al. 1976; Yabe et al. 1997). Recent studies have shown that OSPs, including omission N1 (oN1), omission N2 (oN2), and omission P3 (oP3) components are elicited in response to the omission of self‐generated sensory stimuli (Dercksen et al. 2020, 2024; Ishida and Nittono 2024a; SanMiguel, Saupe, et al. 2013; Stekelenburg and Vroomen 2015).

### 1.1 Predictability of Stimulus Content and OSPs

The predictability of stimulus content has been shown to influence the amplitude of OSPs. When a specific stimulus was presented in response to participants' button press (Dercksen et al. 2020, 2022; Kimura and Takeda 2018; Korka et al. 2020; SanMiguel, Saupe, et al. 2013) or coupled with concurrent stimulus cues, such as a video of a handclap (van Laarhoven et al. 2017), OSPs were larger during stimulus omissions compared to when random stimuli were presented. These findings collectively suggest that the higher the predictability of the stimulus content, the greater the brain response to the omission.

These differences in OSP amplitudes across conditions are often explained using the concept of precision weighting from predictive coding theory (Feldman and Friston 2010; Friston 2009). The gain of sensory units reporting prediction errors is modulated by the precision of the prediction; that is, the precision weights the prediction errors (inverse variance). The more precise the prediction, the higher the sensitivity of the corresponding units, and the greater the prediction errors when that prediction is violated (Barascud et al. 2016; Den Ouden et al. 2012). In previous studies, when predictions about stimulus content were more precise, prediction error signals became larger, leading to larger OSP amplitudes (Bendixen et al. 2012; Braga and Schönwiesner 2022; Heilbron and Chait 2018; Schröger et al. 2015). When the stimulus content was less predictable, the predictions were down‐weighted, leading to smaller OSP amplitudes. Accordingly, OSPs are thought to reflect the content‐specific nature of the sensory prediction (Dercksen et al. 2020; SanMiguel, Saupe, et al. 2013; van Laarhoven et al. 2017). While these studies suggest that the amplitude of OSPs reflects differences in predictability—that is, differences in the precision of predictions about the stimulus content—they provide only indirect evidence that OSPs encode the specific content of the predicted stimulus.

To investigate the specificity of predictions in a musical context, Ishida et al. (2024) compared ERP responses to tone omissions within familiar and unfamiliar melodies. They found that the amplitude of oN1 was greater for omissions in familiar melodies than in unfamiliar melodies, suggesting that higher predictability of upcoming tones increases OSP amplitudes. Furthermore, using machine learning techniques (support vector machine), they successfully decoded the pitch of expected tones, identifying four pitches in familiar melodies with an accuracy of 30.2%—significantly above chance level (25%)—based on scalp potentials from 34 electrodes between 58 and 83 ms post‐tone omission. Since the four tones in the familiar melodies were equally predictable, the decoding cannot be attributed to differences in prediction precision but instead suggests that OSPs contained information about the specific pitch of the predicted stimuli. A similar study using MEG data decoded four omitted tone frequencies and was able to identify them with a peak accuracy of 35% (Demarchi et al. 2019) or < 27% (Hauswald et al. 2024) at 100 ms post‐omission. However, despite being above chance, the differences were modest, and what differences in the scalp potentials or magnetic signals were used in decoding is a black box. As such, it is still not fully understood how specific stimulus features influence predictions and are reflected in prediction error signals.

### 1.2 Modality Differences in OSPs

To address the questions that remain surrounding content‐specific predictions, the present study focuses on sensory modality as a stimulus feature that shows pronounced differences in OSPs. We examined whether predictions about the sensory modality of an expected stimulus modulate the characteristics of OSPs when the stimulus is unexpectedly omitted. Previous research has demonstrated that OSPs vary depending on the sensory modality of the predicted stimulus. A key advantage of using sensory modality stimuli to investigate the content of predictions is that the OSPs of each sensory modality occur with distinct characteristics. It has been consistently reported that auditory OSPs have shorter peak latencies than visual OSPs (Hernández and Hernández‐Sánchez 2017; Ishida and Nittono 2024a; Nittono 2005; Simson et al. 1976). This latency difference is thought to result from the faster transduction speed of sound in hair cells compared to the phototransduction speed in the retina (King 2005; Torre et al. 1995). This distinction enables more direct inferences regarding the cause of the observed differences in OSPs.

While previous findings suggest that omitted stimuli are processed through modality‐specific neural pathways, these studies have presented auditory and visual stimuli in separate blocks. When stimuli are segregated by block, factors unrelated to sensory modality—such as differences in motor response timing or overall arousal and attention levels—may influence the results. To overcome this limitation, the present study introduced auditory and visual stimuli within the same block to examine whether OSPs occur differently depending on the sensory modality of the predicted stimuli. This approach allowed us to ensure that the differences in OSPs across trials, where stimuli of different sensory modalities were predicted, reflect modality‐specific predictions.

### 1.3 Current Study

In the present study, participants were asked to press a mouse button at a constant interval, with each press triggering either an auditory or visual stimulus. Auditory and visual stimuli were presented twice in alternating order. Using voluntary button presses to present stimuli has become a common method for investigating OSPs (Dercksen et al. 2020, 2022, 2024; Ishida and Nittono 2024a; Kimura and Takeda 2018; Korka et al. 2020; Nittono 2005; Nittono and Sakata 2009; SanMiguel, Saupe, et al. 2013; SanMiguel, Widmann, et al. 2013; Stekelenburg and Vroomen 2015). This paradigm is advantageous for OSP recordings, as it provides a clear time‐locking reference, which can reduce latency jitter in early responses (Schröger et al. 2015). Voluntary actions generate the efference copy propagated from the motor area to sensory processing areas, where the consequent sensation is expected (Crapse and Sommer 2008), which enhances the brain's ability to predict the timing of the stimulus compared to passive stimulus presentation. This is particularly important in OSP measurements, where the absence of a physical cue (i.e., deviant stimulus) for deviance detection can lead to latency jitters of brain responses, potentially obscuring clear waveforms.

While prior studies have employed different types of stimuli triggered by button presses, they have fixed the sensory modality within each block. By presenting auditory and visual stimuli twice alternately within the same block and omitting stimuli with equal probability at each position, we ensured that the probability of each modality preceding an omission was equal. This procedure has been shown to effectively control the influence of evoked potentials from the preceding stimulus on the stimulus‐absent epoch (Ishida and Nittono 2024b).

We recorded ERPs for these omissions and compared the resulting OSPs between the sensory modalities of predicted stimuli. If the predictions were modality‐specific, the peak latencies of OSPs elicited by trials in which auditory stimuli were expected (auditory omission trials) would be shorter than those elicited by trials in which visual stimuli were expected (visual omission trials) (H1). Furthermore, we explored differences in OSP topographies that would differ between sensory modalities to see if distinct cortical regions are involved in processing modality‐specific prediction errors (H2). If confirmed, these findings would provide evidence that people predict upcoming stimulus content in specific ways according to sensory modality and that violations of these predictions elicit qualitatively different OSPs.

## 2 Method

### 2.1 Participants

We used a sample size comparable to that used in Ishida and Nittono (2024a), which examined the OSP latency differences between visual and auditory blocks (*N* = 29). We recruited 40 undergraduate and graduate students, considering the potential for data exclusion. All participants self‐reported having normal vision and hearing. Written informed consent was obtained prior to the experiment. Participants received a cash voucher of 2500 Japanese yen as an honorarium. The study protocol was approved by the Behavioral Research Ethics Committee of the Osaka University School of Human Sciences, Japan (H024‐014) in accordance with the Declaration of Helsinki. Following the application of the exclusion criteria detailed below, data from 33 participants (18 males and 15 females, 18–33 years old, *M* = 21.7 years old) were used for the analysis. All but one were right‐handed (FLANDERS handedness questionnaire; Okubo et al. 2014).

### 2.2 Stimuli

A 1000‐Hz pure tone (16‐bit quantization, 48‐kHz A/D sampling) was presented for 70 ms (rise/fall 10 ms) as an auditory stimulus. An LED light (45 mm × 86 mm) was presented for 66.7 ms as a visual stimulus. Before the experiment, participants were asked to adjust the tone's volume to match the light intensity (approximately 112.0 cd/m^2^) so that the perceived intensities of the light and tone would be subjectively equal. The LED light (white LED backlight module, Product ID 1621, Adafruit, USA) was placed on a tabletop in front of the participant at a viewing distance of 44 cm, with the center of the LED positioned at a height of 32.3 cm above the tabletop. The light was slightly lower than the participant's eye level. Since it was presented at central fixation, the height was not adjusted for individual participants. To minimize the influence of ambient lighting on the participant's vision, the visual stimuli were enclosed within a three‐sided partition surrounding the LED light. Auditory stimuli were presented binaurally through canal‐type (closed‐type) earphones (MDR‐EX650AP; SONY, Tokyo, Japan) using the Ez‐SOUND sound stimulus system (Nihon Santeku, Osaka, Japan). The experiment was controlled using Inquisit 6.5.1 (Millisecond Software; Seattle, WA, USA). For stimulus presentation, a TTL signal was sent from the parallel port to trigger the dedicated auditory stimulation device (Ez‐Sound) and to activate the LED to minimize delay and latency jitter. The timing between the TTL signal and actual stimulus presentation was verified at the millisecond level using an oscilloscope. This verification confirmed that auditory and visual stimuli were presented simultaneously (within 1 ms) with the trigger and thus both stimuli were presented at the same timing.

### 2.3 Procedure

**FIGURE 1:**
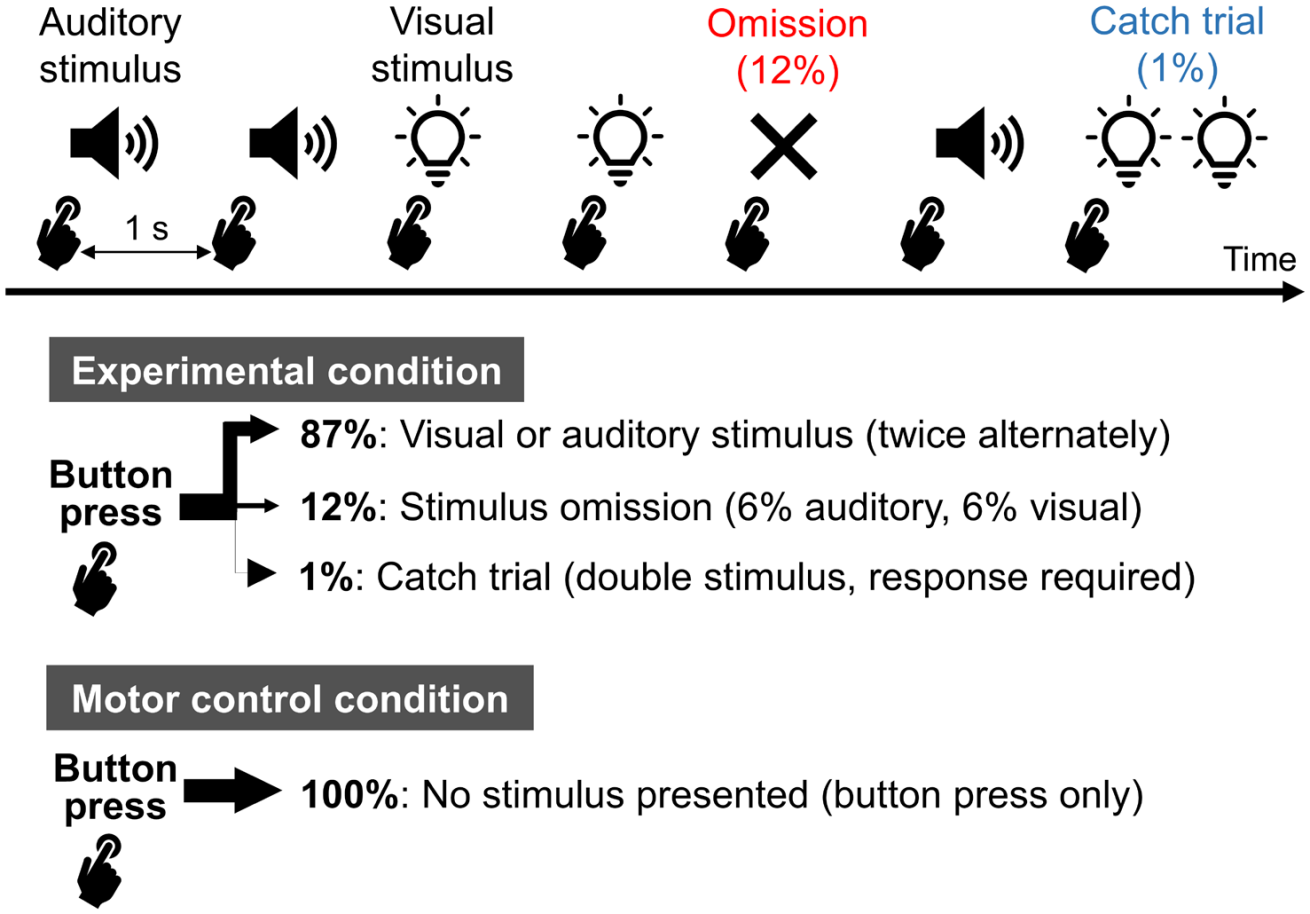
Schematic representation of the experimental design. Participants pressed a mouse button at intervals of approximately 1 s. Immediately after each button press, an auditory stimulus (1000 Hz pure tone) or a visual stimulus (LED light) was presented. The auditory and visual stimuli were presented twice each in an alternating sequence, with one stimulus presented per button press. Stimuli were omitted in 12% of the trials. Participants were instructed to respond with the opposite hand during catch trials, in which a tone or light was presented twice following a single button press. In the motor control condition, no stimuli followed button presses.

Figure 1 illustrates a schematic representation of the experiment. Participants were asked to press a wired mouse button (M‐U0025‐O; NEC, Tokyo, Japan) with their index finger at a constant interval of 1 s. For each button press, either an auditory or a visual stimulus was presented once. Auditory and visual stimuli alternated, with two consecutive presentations of each modality (e.g., A‐A‐V‐V‐A‐A‐V‐V…), and a rare stimulus omission (*p* = 0.12) occurred at each position in the sequence (if the auditory or visual stimulus was omitted, it was counted as one of the two presentations for that modality). This ensured that the sensory modality of the stimulus preceding the omission occurred with equal probability. The first four trials of each block, as well as the four trials following an omission trial, were always stimulus trials. The hands used for button pressing were counterbalanced across the participants. The experiment consisted of 300 trials per block (36 omission trials and 264 stimulus trials (including catch trials), excluding the first four fixed stimulus trials), and participants completed eight blocks for a total of 2400 trials. Thus, each sensory modality had 1056 stimulus trials and 144 omission trials for analysis.

Participants were instructed to fixate on the LED light throughout the task, and a chin rest was used to minimize head movement. To ensure fixation, participants were asked to respond to catch stimuli (i.e., two consecutive auditory or visual stimuli were presented with an interstimulus interval of 70 ms) by pressing a button with the thumb of the opposite hand. Catch trials occurred 2–3 trials per block (approximately 0.8%), with auditory and visual catch trials appearing to be equiprobable overall throughout the experiment. At least four trials after the catch trial were always stimulus trials. Late responses (more than 1000 ms after the onset of the catch trial) or no responses during the catch trials were recorded as catch trial errors.

The stimulus was presented immediately after each button press if it occurred within 600–1400 ms after the previous button press. If the button was pressed outside this range (i.e., too early or too late), no stimulus was presented. This was counted as a button press error and not classified as an omission trial. Participants were informed that occasionally a stimulus would not be presented even after a correct button press. Thirty practice trials were provided at the beginning of the experiment to help participants become familiar with the appropriate button press interval. At the beginning and end of the experiment, participants were asked to press the button 80 times at a regular 1‐s interval without receiving any stimulus (160 times in total), which served as the motor control condition. The data from this control condition were used to correct for movement‐related potentials in the ERP waveforms of the experimental condition (auditory and visual omission trials). The entire experiment took approximately 2 h, including electrode preparation and short breaks between blocks.

### 2.4 EEG Data Recording

EEG data were recorded from 64 sites using Ag‐AgCl active electrodes with the ActiveTwo system (BioSemi, Netherlands), at a sampling rate of 2048 Hz and a bandpass filter of 0–400 Hz. A reference electrode (Common Mode Sense [CMS], active electrode) and a ground electrode (Driven Right Leg [DRL], passive electrode) were also attached to the scalp. Additional electrodes were placed on the nose tip for offline re‐referencing, as well as on the left and right outer canthi of the eyes and above and below the right eye for vertical and horizontal electrooculograms, using UltraFlat active electrodes.

### 2.5 Exclusion Criteria

Seven participants were excluded from the analysis based on the following criteria: 44 or more rejected omission trials (30% of the 144 omission trials) due to artifacts (*n* = 7), 10% or more button press errors in the experimental condition (*n* = 1, already excluded by the first criterion), and 70% or fewer hits and/or 15% or more false alarms in the catch trials (*n* = 0). As a result, data from 33 participants were included in the analysis. For these, the mean percentages of button press error trials were 1.07% in the experimental condition and 2.01% in the motor control condition. These button press error trials were excluded from further analysis. For the catch trials, the mean hit rate was 94.55%, and the mean false alarm rate was 0.59%. Catch trials, trials immediately following catch trials, and trials containing a catch trial response (false alarm response) in the period of 1400 ms before and 500 ms after a stimulus or an omission were also excluded.

### 2.6 EEG Data Reduction

The EEG data were analyzed using Brain Vision Analyzer 2.2.2 (Brain Products, Germany). The data were resampled to 512 Hz, a digital bandpass filter of 0.1–30 Hz was applied, and eye blinks and eye‐movement‐induced artifacts were corrected using Gratton et al.'s (1983) method. These settings were the same as those of Ishida and Nittono (2024a). Epochs were averaged separately from 200 ms before to 500 ms after the button press for each modality. Epochs with voltages exceeding ±250 μV were removed (Foti et al. 2009; Ishida and Nittono 2024a). The mean rejection rates were 10.77% for the auditory omission trials, 9.87% for the visual omission trials, and 7.27% for the motor control condition. Baseline correction was applied by subtracting the mean amplitude of the initial 200‐ms period from each point of the waveform. The ERP waveforms of the motor control condition were subtracted from those of the experimental condition to correct for movement‐related potentials.

Peak latencies were identified for each participant on the motor‐corrected waveforms at T8 (Ishida and Nittono 2024a; Nittono 2005) as the most negative peak within the time window of 50–150 ms for oN1, and 150–250 ms for oN2 for both the auditory and visual modalities. Time windows were determined based on previous studies (Ishida and Nittono 2024a; Nittono 2005) and visual inspection of the grand mean difference waveforms. Peaks were detected using an automated local minimum search algorithm and then visually inspected. Based on previous studies (Dercksen et al. 2020; Ishida and Nittono 2024a; Nittono 2005; Nittono and Sakata 2009; SanMiguel, Saupe, et al. 2013) and visual inspection of the scalp topographies, two regions of interest (ROIs) were defined for analysis: frontocentral (F1, Fz, F2, FC1, FCz, FC2, C1, Cz, and C2) and right temporal (FC4, FC6, FT8, C4, C6, T8, CP4, CP6, and TP8). The oN1 amplitude was quantified as the mean voltage in a 20‐ms interval centered on the mean peak latency across participants in the frontocentral ROI. The oN2 amplitude was quantified as the mean voltage in a 40‐ms interval centered on the mean peak latency across participants in the right temporal ROI. Since oP3 was not clearly identifiable and its peak could not be determined using the same procedure as for oN1 and oN2 in either the auditory or visual modality, the time windows of 250–400 ms for the auditory modality and 300–450 ms for the visual modality were used in the frontocentral ROI, based on the time windows for oP3 used in Ishida and Nittono (2024a).

### 2.7 Statistical Analysis

A two‐tailed paired *t*‐test was performed to compare the mean button press intervals in the experimental and motor control conditions. To examine the presence of prediction‐related responses, one‐tailed one‐sample *t*‐tests against zero were performed separately on the mean amplitudes of the difference waveforms for the auditory and visual omission trials in their respective time windows and corresponding regions of interest, for each of oN1, oN2, and oP3. To examine the differences in peak latencies between sensory modalities (H1), one‐tailed paired *t*‐tests were performed for the oN1 and oN2 peak latencies. Corresponding Bayesian *t*‐tests were conducted to evaluate the evidence for either no difference (effect size δ = 0, null hypothesis) or the presence of a difference (alternative hypothesis: effect size δ ≠ 0 for two‐tailed, δ < 0 or δ > 0 for one‐tailed, depending on the expected direction), using JASP version 0.19.0 (JASP Team 2024). For the frequentist approach, the significance level was set at 0.05. For the Bayesian analysis, the prior distribution for δ was modeled using a Cauchy distribution with a scale parameter of *r* = 0.707. Resulting Bayes factors (BF_10_ for two‐tailed, BF_−0_ or BF_+0_ for one‐tailed) > 3 were interpreted as moderate evidence supporting the alternative hypothesis and less than 0.33 were interpreted as moderate evidence supporting the null hypothesis based on Schönbrodt and Wagenmakers (2018). Note that BF_−0_ or BF_+0_ indicates the Bayes factor comparing a directional alternative hypothesis (δ < 0 or δ > 0, respectively) against the null hypothesis (δ = 0), as described by Wagenmakers et al. (2018).

To test whether significant differences in scalp topographies existed between the auditory and visual omission trials (H2), a topographical analysis of variance (TANOVA) was conducted using Ragu software (Habermann et al. 2018; Koenig et al. 2011). TANOVA is a non‐parametric randomization test that quantifies the dissimilarity between two scalp potential maps (Murray et al. 2008). First, Global Field Power (GFP) was computed for each map as the standard deviation of the voltage values across all electrodes at a given time point. Each scalp potential map was then normalized by dividing the voltage at each electrode by the GFP at that time point, ensuring that comparisons focused on spatial patterns rather than absolute signal strength (Koenig et al. 2011). Topographic differences between conditions were then quantified using dGFP, a global measure of scalp field differences computed as the root mean square deviation of the condition‐wise grand mean voltages from the overall grand mean across all conditions and electrodes (Habermann et al. 2018; Koenig et al. 2011). This approach allows for an examination of differences in topographic patterns (spatial distributions) while controlling for absolute signal strength.

To identify the latency ranges with significant topographic differences, TANOVA does not require a priori assumptions about time windows or electrode locations (Koenig et al. 2011). A randomization test with 5000 permutations was performed at each time point to generate a null distribution to estimate the probability of obtaining the observed topographic differences by chance. To address the issue of multiple comparisons across time points, Global Duration Statistics implemented in Ragu was applied (Habermann et al. 2018; Koenig et al. 2011). This method evaluates the duration of consecutive significant time points by generating a null distribution of the longest randomly occurring significant clusters. Only clusters exceeding the 95th percentile of this distribution were considered statistically significant. In the present study, the scalp topographies of OSPs in the visual and auditory conditions before subtracting the motor control condition were used for the comparison. TANOVA indicated that a cluster of significant time points must persist for at least 37 ms to be considered statistically reliable. Any shorter duration of significance was discarded as likely due to chance.

## 3 Results

### 3.1 Behavioral Results

The mean interval of button presses from preceding button presses was shorter in the experimental condition (*M* = 815 ms, SD = 112 ms) than in the motor control condition (*M* = 937 ms, SD = 112 ms), *t*(32) = −8.72, *p* < 0.001, *dz* = −1.52, BF_10_ > 100.

### 3.2 ERPs

**FIGURE 2:**
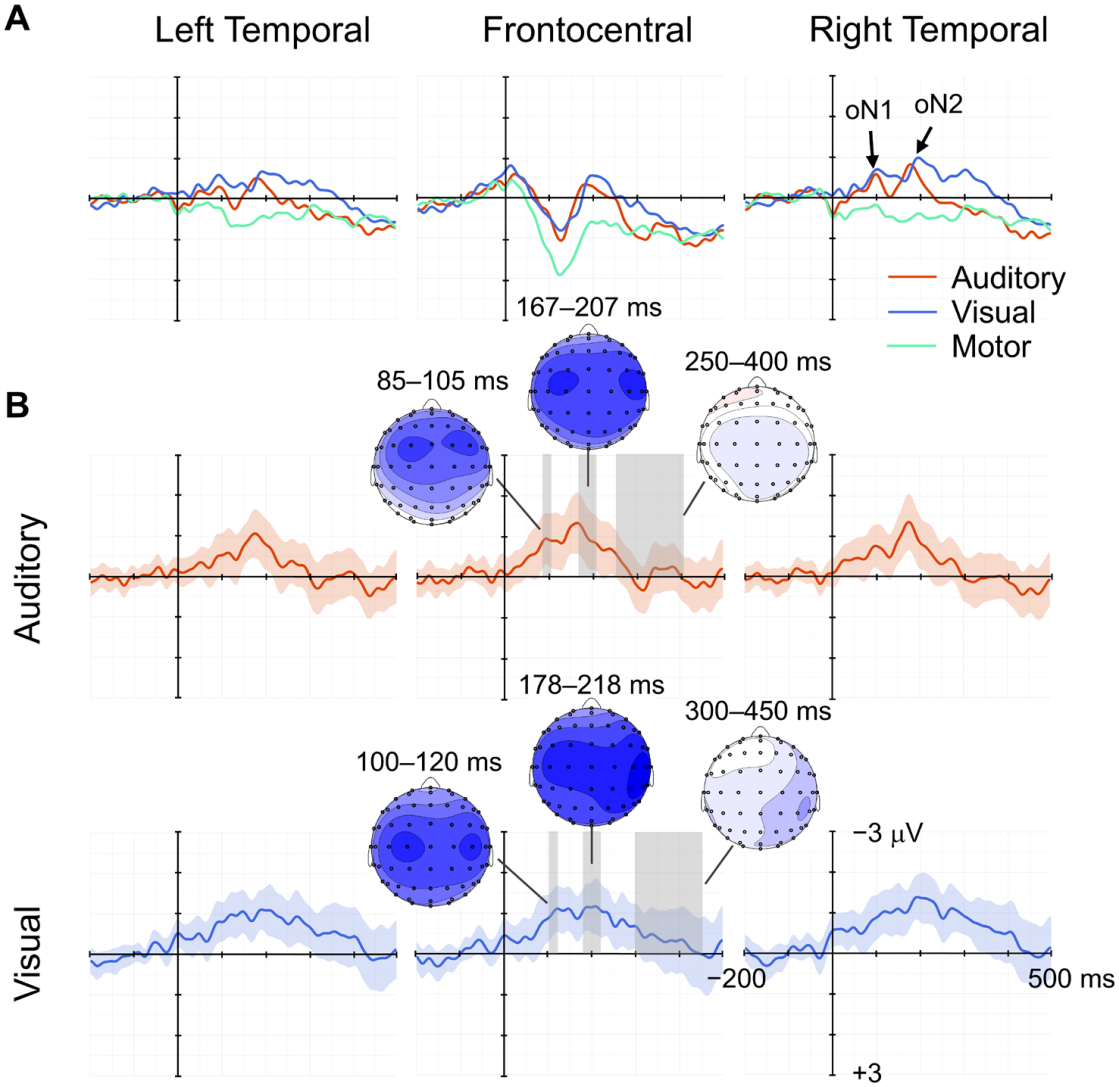
Grand mean ERP waveforms and difference waveforms (motor‐corrected) of omission trials. (A) Grand mean ERP waveforms of the auditory omission trials (red), visual omission trials (blue), and motor control condition (green), averaged across left temporal electrodes (FC3, FC5, FT7, C3, C5, T7, CP3, CP5, and TP7), frontocentral electrodes (F1, Fz, F2, FC1, FCz, FC2, C1, Cz, and C2), and right temporal electrodes (FC4, FC6, FT8, C4, C6, T8, CP4, CP6, and TP8). (B) Motor‐subtracted difference waveforms of the auditory and visual omission trials, each plotted with 95% confidence intervals. Scalp topographies, derived from motor‐subtracted difference waveforms of the auditory and visual omission trials in oN1, oN2, and oP3 time windows, are overlaid on the waveforms.

Figure 2 shows the grand mean and difference ERP waveforms for the auditory and visual omission trials and the motor control condition, as well as the motor‐corrected scalp topographies of the mean amplitudes in the oN1, oN2, and oP3 time windows. Corresponding ERP waveforms for stimulus trials are shown in the Figure S1. Motor‐related positive potentials were predominant in the frontocentral region around the 100–200 ms interval, which were superimposed by deflections corresponding to the oN1 and oN2 components in both modalities. oN1 was predominant in the frontocentral region, and oN2 was predominant in the right temporal region in both modalities. In both components, the visual modality showed a more posterior and lateral distribution than the auditory modality. oP3 was absent in the waveforms and topographies in both sensory modalities. This was also true for the centroparietal ROI, where oP3 (P300) is normally predominant (see the Figure S2).

The oN1 amplitude was quantified as the mean voltage of 85–105 ms time window for the auditory modality, and 100–120 ms for the visual modality in the frontocentral ROI. The oN1 amplitudes were significantly negative from baseline for both the auditory (*M* = −0.90 μV, SD = 1.77 μV) and visual trials (*M* = −0.98 μV, SD = 1.55 μV), *t*(32) = −2.92, *p* = 0.003, *dz* = −0.51, BF_−0_ = 12.81; *t*(32) = −3.64, *p* < 0.001, *dz* = −0.63, BF_−0_ = 67.04, respectively. The oN2 amplitude was quantified as the mean voltage of 167–207 ms time window for the auditory modality, and 178–218 ms for the visual modality in the right temporal ROI. The oN2 amplitudes were significantly negative from baseline for both the auditory (*M* = −1.13 μV, SD = 1.82 μV) and visual trials (*M* = −1.34 μV, SD = 1.54 μV), *t*(32) = −3.57, *p* < 0.001, *dz* = −0.62, BF_−0_ = 57.05; *t*(32) = −4.99, *p* < 0.001, *dz* = −0.87, BF_−0_ > 100, respectively. The oP3 amplitudes were quantified as the mean voltage of 250–400 ms time window for the auditory modality, and 300–450 ms for the visual modality in the frontocentral ROI. The amplitudes of the oP3 time window were not significantly different from baseline for either the auditory (*M* = −0.12 μV, SD = 1.40 μV) and the visual modality (*M* = −0.32 μV, SD = 1.91 μV), *t*(32) = −0.51, *p* = 0.694, *dz* = −0.09, BF_+0_ = 0.13; *t*(32) = −0.96, *p* = 0.827, *dz* = −0.17, BF_+0_ = 0.10, respectively.

**FIGURE 3:**
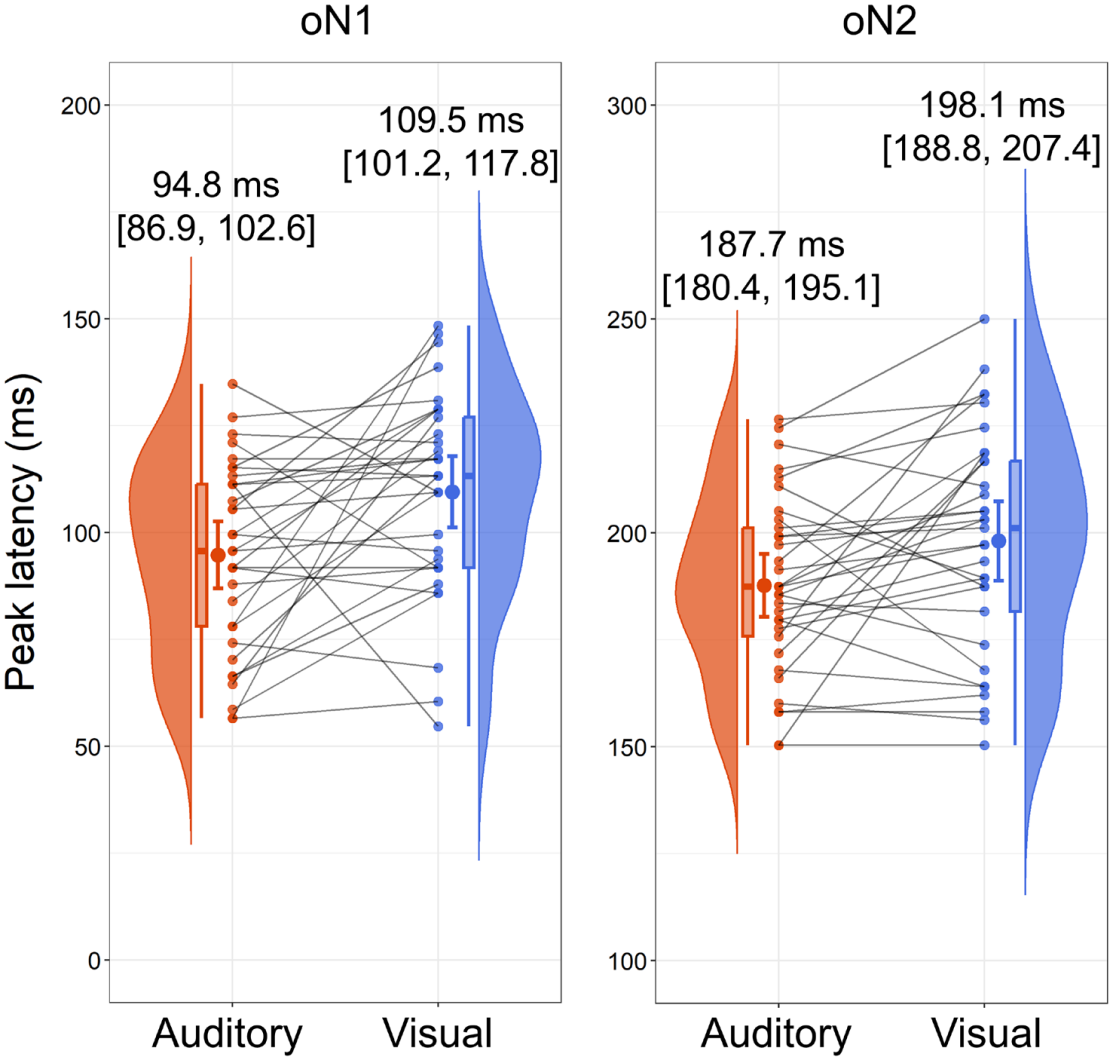
Comparisons of oN1 and oN2 peak latencies between the auditory and visual omission trials. In both modalities, the peak latencies of oN1 were identified within a 50–150 ms time window, and the peak latencies of oN2 were identified within a 150–250 ms time window. Above the raincloud plots, the mean peak latencies are shown with their 95% confidence intervals.

Figure 3 shows the results of peak latency comparisons for oN1 and oN2 between modalities. The oN1 peak latency was significantly shorter in the auditory omission (*M* = 94.8 ms, SD = 22.1 ms) than in the visual omission trials (*M* = 109.5 ms, SD = 23.5 ms), *t*(32) = −2.96, *p* = 0.003, *dz* = −0.52, BF_−0_ = 13.93. Similarly, the oN2 peak latency was significantly shorter in the auditory omission (*M* = 187.7 ms, SD = 20.8 ms) than in the visual omission trials (*M* = 198.1 ms, SD = 26.1 ms), *t*(32) = −2.57, *p* = 0.007, *dz* = −0.45, BF_−0_ = 6.17.

As suggested by the reviewers, the button press intervals, OSP amplitudes, and OSP peak latencies were analyzed separately for each sequence position (i.e., A‐A‐V‐V) and are reported in the Supporting Information S1. The OSP results were generally consistent with the main analyses described above.

**FIGURE 4:**
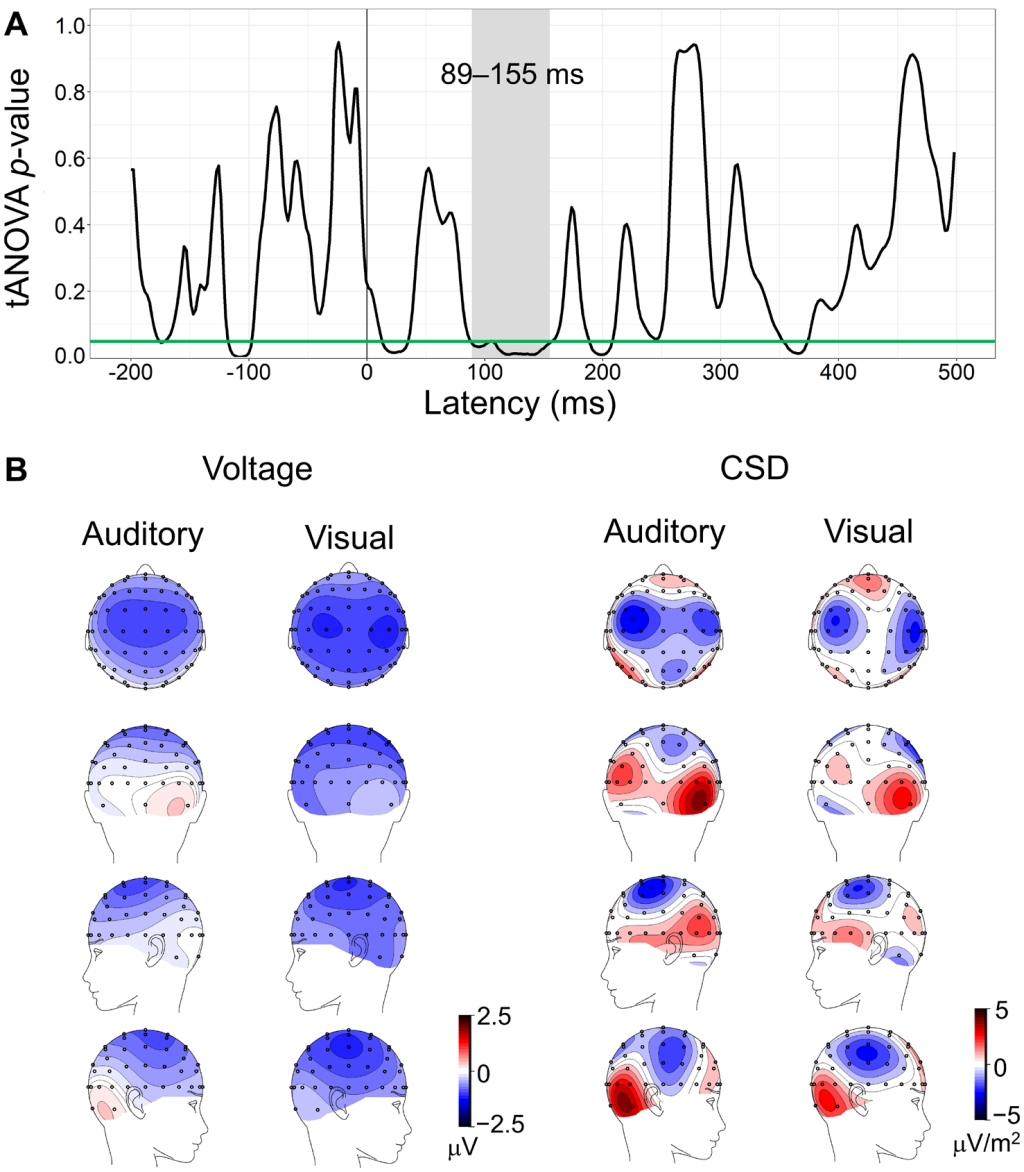
Comparisons of topographies between the auditory and visual omission trials. (A) The results of the TANOVA comparing scalp topographies for the auditory and visual omission trials. The line depicts the TANOVA's point‐by‐point *p*‐values, with the green line indicating *p* = 0.05. The shaded area highlights the time window of reliable differences between the modalities. (B) Scalp voltage and CSD maps plotted from motor‐subtracted difference waveforms for the auditory and visual omission trials in the time window of reliable differences (89–155 ms).

Figure 4 shows the results of the TANOVA and the scalp topographies in the time window with reliable differences. The scalp topographies differed significantly between the auditory and visual omission trials in the 89–155 ms interval. The shortest duration of significant effect was 37 ms, as determined by Global Duration Statistics, confirming that the observed 66 ms difference (i.e., 89–155 ms) was not due to random fluctuations. The scalp voltage and current source density (CSD) maps in the visual omission showed a slightly more posterior and lateral distribution than the auditory omission, similar to the patterns observed in the oN1 and oN2 time windows.

## 4 Discussion

In this study, we examined brain responses to the unexpected omission of auditory and visual stimuli within a context in which the sensory modality of stimuli triggered by participants' voluntary actions was predictable. The oN1 and oN2 components were observed in both sensory modalities. oN1 and oN2 had clear peaks in both sensory modalities, with comparable amplitudes for auditory and visual OSPs. The peak latencies for oN1 and oN2 were significantly shorter in the auditory omission trials than in the visual omission trials (supporting H1). Furthermore, the topographies of OSPs differed significantly between sensory modalities (supporting H2). In the oN1 and oN2 time windows, the negative potentials had more central and frontal distributions for auditory omissions than for visual omissions. These findings align with those of Ishida and Nittono (2024a), who presented the auditory and visual stimuli in separate blocks.

The peak latencies of oN1 and oN2 were shorter for auditory omissions than for visual omissions, consistent with the results of previous research (Hernández and Hernández‐Sánchez 2017; Ishida and Nittono 2024a; Nittono 2005; Simson et al. 1976). This may be because the time required for signals to travel from the sensory organ to the sensory cortex is shorter for auditory signals than for visual signals (King 2005; Torre et al. 1995). The current findings suggest that even within the same block, the brain recruits neural pathways specific to the predicted sensory modality.

Statistically significant differences in scalp topographies were observed between the auditory and visual omission trials during the 89–155 ms time window, which corresponds to the whole range of oN1 and the rising phase of oN2. In this time window, as in the time window around the peak latencies of oN1 and oN2, negative potentials were predominant over the central and frontal scalp regions in the auditory omission, while they were more posteriorly and laterally distributed in the visual omission. Thus, the predicted sensory modality affects the scalp topography of OSPs.

oN1 is considered to reflect prediction error, the discrepancy between the predicted and the actual inputs, and oN2 is interpreted as a sign of higher order error processing (SanMiguel, Saupe, et al. 2013; van Laarhoven et al. 2017). The differences in scalp distributions in the present study indicate that distinct neural generators responsible for processing prediction errors were involved according to the sensory modality of the predicted stimulus. This result is consistent with previous research on mismatch negativity (MMN), which has shown that in a multimodal context, the brain adjusts sensory regions to process the deviance according to the regularity of the stimulus sequence. Grundei, Schmidt, et al. (2023) and Grundei, Schröder, et al. (2023) demonstrated that when a regularity involving stimulus intensity was violated in one sensory modality in a paradigm presenting auditory, visual, and somatosensory stimuli simultaneously, MMN was elicited in brain regions specific to the modality of the deviating stimulus. In the present study, we evaluated differences in neural responses under identical conditions in which physical input was absent, making our findings a more direct indication that different processing occurs depending on the predicted sensory modality.

In the present study, we presented a single sequence consisting of stimuli from two sensory modalities. Rather than a simple prediction in which a unimodal stimulus is merely repeated, the formation of an intermodal prediction—where two sensory modality stimuli alternated twice—likely required the involvement of higher‐order regions that integrate information from each sensory cortex. This may have led to the predominance of processing in higher‐order sensory areas, resulting in a more anterior distribution of visual oN1 and oN2 compared to previous visual OSP studies, including Ishida and Nittono (2024a), all of which employed unimodal sequences. In Grundei, Schröder, et al. (2023), vMMN in response to deviations of visual stimuli within a multimodal sequence showed a more temporal distribution compared to the classic occipital‐dominant vMMN. This corresponds to the distribution of visual oN1 and oN2 in the present study.

The finding that early‐to‐mid OSPs occurred in a modality‐specific manner supports the framework of hierarchical predictive coding theory. This framework posits that prediction errors are compared with templates within the predicted sensory cortices, and as information ascends the hierarchy, it becomes progressively integrated, enabling the processing of multimodal events (Arnal and Giraud 2012; Friston 2005; Friston and Kiebel 2009; Rao and Ballard 1999). In the present study, prediction errors generated in the auditory and visual cortices were likely propagated to higher‐order areas, where they were integrated within the sequence of alternating auditory and visual stimuli, allowing the omissions to be interpreted as deviations from this structured sequence. Furthermore, this study reinforces findings on the feature‐specific nature of predictions by presenting stimuli from two sensory modalities within a single sequence, thereby controlling for confounding factors such as arousal level, attention level, and motor response timing. While most previous OSP studies have demonstrated that OSPs vary quantitatively in response to differences in the predictability or precision of the predicted stimulus, according to the precision weighting prediction errors (Feldman and Friston 2010; Friston 2009), the current findings extend this by showing that differences in the predicted stimulus content, specifically the sensory modality, lead to qualitatively distinct OSPs. This suggests that predictions go beyond simply reflecting a gradient of precision and instead encompass specific information about the physical characteristics of the expected stimulus.

Empirical evidence on the specificity of predictions about stimulus features remains insufficient; however, research employing stimulus omission paradigms is accumulating. Ishida et al. (2024) demonstrated that expected sound pitch information is reflected in oN1. Additionally, Ishida and Nittono (2024b) showed that visual MMN was more prominent for omissions in the lower visual field than for those in the upper visual field when a stimulus was alternately presented twice in each field and was occasionally omitted, suggesting that OSPs may reflect the spatial location of the predicted stimulus. Similar findings, in which the omission of a specific stimulus led to neural responses that reflected the sensory characteristics of the expected input, have also been reported in fMRI (Berlot et al. (2018): tone frequency; Kok et al. (2014): grating orientation) and MEG studies (Demarchi et al. 2019; Hauswald et al. (2024): tone frequency). By using sensory modality stimuli, this study adds to previous findings on the specificity of predictions, showing that differences in expected sensory modalities resulted in clearly distinct OSPs.

Unlike Ishida and Nittono's (2024a) study, in which stimulus omissions were task relevant, oP3 was not observed in this study. In addition to the randomly assigned 12% omission trials, no stimulus was presented when the button press interval was excessively long or short (i.e., error trials). Therefore, participants' button press performance was at least partially associated with the presence or absence of stimuli in the present study. This discrepancy, despite the task relevance of the omissions, may be due to differences in experimental settings, such as whether the experimental block included omissions in a single sensory modality (previous studies) or omissions in multiple sensory modalities (the present study). Further research is needed to clarify the experimental settings under which oP3 emerges.

### 4.1 Limitations

This study has several limitations. While catch trials were included in the experimental condition, they were not included in the motor control condition. This means that there may have been some unintended differences between the experimental and motor control conditions in the motor action itself. For instance, the mean interval between button presses was longer in the motor control condition (*M* = 937 ms) than in the experimental condition (*M* = 816 ms). However, the use of auditory and visual stimuli within the same block ensured that any motor‐related effects were equal across modalities. Therefore, the differences in OSPs observed between the auditory and visual omission trials can be attributed to modality‐specific processing rather than differences in motor preparation or execution between the experimental and motor control conditions.

Second, because stimulus omissions were used as feedback for button press errors, the average omission probability reached approximately 13%, with the highest error‐rate participant exhibiting a probability of approximately 19%. Variability in omission probabilities across participants may have introduced unintended effects. However, in an ex post analysis, button press error rates did not significantly differ between sensory modalities (see Table S1). Therefore, the observed differences in OSPs between sensory modalities are unlikely to be attributable to this factor. In addition, it is possible that the absence of a stimulus following a button press may have signaled the participant's poor performance, thereby involving performance monitoring processes. In such cases, ERP components such as the error‐related negativity (ERN) or feedback‐related negativity (FRN) may have been elicited in omission trials. However, Ishida and Nittono (2024a), using a similar omission paradigm, found that an FRN‐like component appeared only when participants were explicitly instructed that stimulus presentation depended on their correct performance. In contrast, when they were instructed that omissions occurred randomly—as in the present study—no FRN was observed, even though stimuli were similarly omitted when the button press interval was excessively long or short. The scalp topographies for omission trials in the present study do not show a frontocentral focus typically associated with the FRN or ERN. Even if performance monitoring processes were involved to some extent, they were likely present in both the auditory and visual trials.

Third, the use of the mouse button may not have been optimal because the clicks and tactile stimulation were also present in the omission trials. In future studies, the use of a non‐contact infrared switch may help to minimize auditory and tactile stimulation. However, even in this case, the proprioceptive sensation cannot be eliminated. In contrast, the subtraction method can cancel out any physical/exogenous effects of the mouse click and extract endogenous components associated with stimulus omission.

Fourth, it remains unclear whether the current findings can be generalized to OSPs recorded in passive paradigms without self‐initiated actions triggering stimuli. N1 and P2 are known to be attenuated in response to stimuli generated by self‐initiated actions (Knolle et al. 2013), and when those self‐generated stimuli deviate from expectations, N2 and P3 are enhanced compared to the same deviations in automatically presented stimuli (Knolle et al. 2013; Nittono 2006). These findings suggest that the neural mechanisms involved may differ depending on whether the stimulus is a consequence of one's own actions. Further research is needed to explore this possibility.

Fifth, the nature of the predictions formed during the task—whether they were automatic or controlled and conscious—remains unclear. Although this experiment repeated a fixed stimulus sequence, future research should examine whether OSPs corresponding to the expected sensory modality would similarly occur when randomly presented cues indicate the upcoming sensory modality, or when participants themselves anticipate the modality of stimuli presented in a random order.

## 5 Conclusion

The present study demonstrates that predictions about the content of stimuli are formed, resulting in content‐specific OSPs even within the same context. Our results show that when predictions are violated, distinct neural responses are elicited depending on the predicted sensory modality. This suggests that the brain forms modality‐specific predictions about upcoming stimuli and that OSPs reflect such prediction content. By revealing how modality‐specific predictions are reflected in OSPs, this study offers insight into the mechanisms by which the brain flexibly adapts to a multisensory environment.

## Author Contributions

**Tomomi Ishida:** conceptualization, data curation, formal analysis, investigation, methodology, project administration, visualization, writing – original draft. **Hiroshi Nittono:** conceptualization, methodology, writing – review and editing.

## Conflicts of Interest

The authors declare no conflicts of interest.

## Supporting information


Data S1.

## Data Availability

The datasets analyzed for the present paper are available at https://osf.io/7ck9v/.
