# Supplementary material for: Predicted Sensory Modality Determines the Timing and Topographies of Omitted Stimulus Potentials
Source: Psychophysiology. 2025 Jun 25;62(6):e70097. doi: 10.1111/psyp.70097 (PMC12188284; doi:10.1111/psyp.70097)
Supplement: Supplementary file 1 — Data S1. [file PSYP-62-e70097-s001.pdf]

Supporting Information

Predicted Sensory Modality Determines the Timing and Topographies of Omitted Stimulus Potentials

ERPs to Physical Stimuli

Figure S1 shows the ERP waveforms and topographies for the auditory and visual stimulus trials. Similar to the ERPs for omissions, the peak latencies of N1 and P2 were shorter in the auditory stimulus trials than in the visual stimulus trials. In the auditory stimulus trials, the topography of the N1 time window shows the frontocentral distribution. In the visual stimulus trials, the topography of the N1 time window shows the temporal distribution, whereas the topographies of pre-N1 and P2 time windows show the occipital distribution.

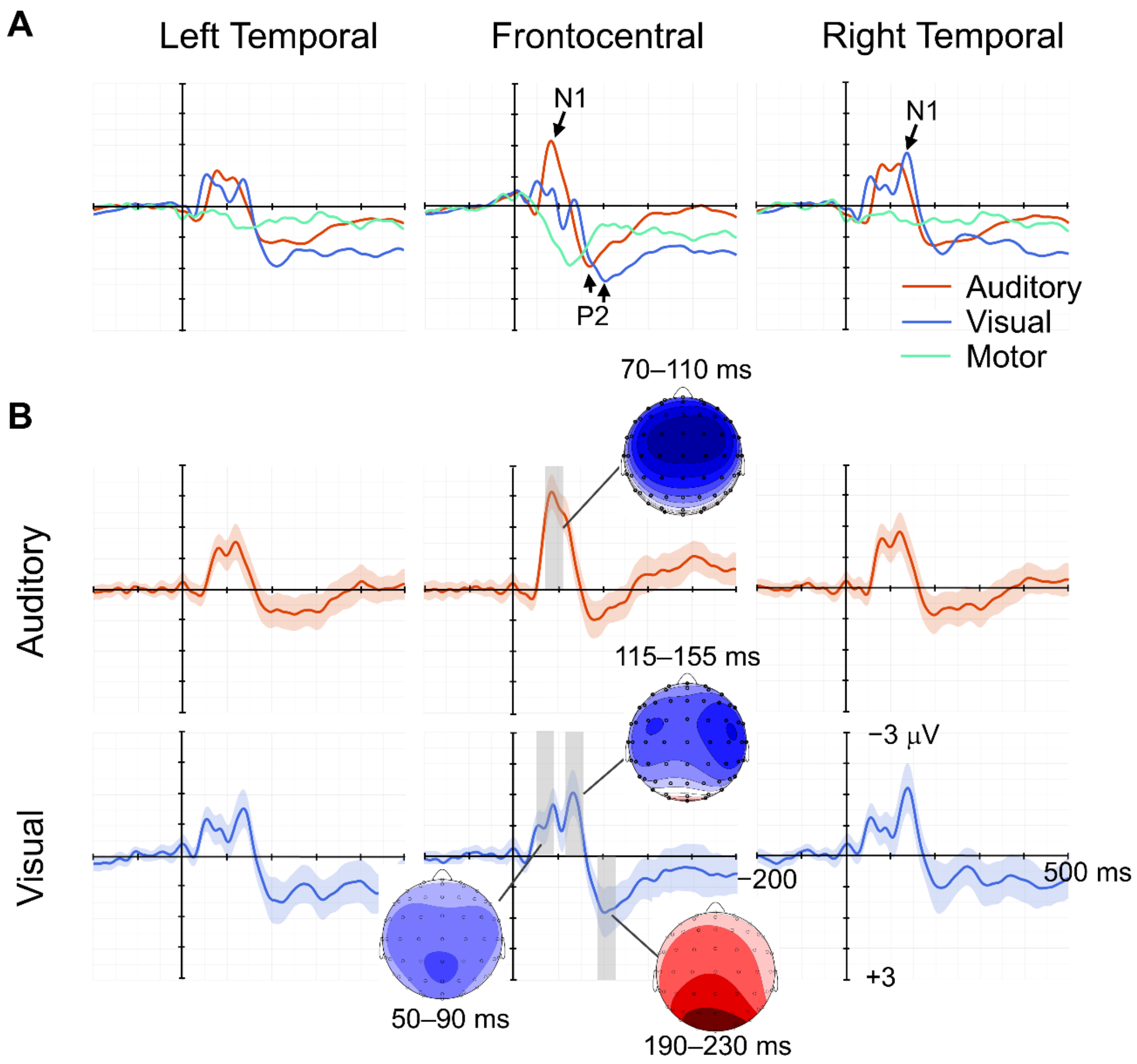

FIGURE S1. Grand mean ERP waveforms and difference waveforms (motor-corrected) of stimulus trials. (A) Grand mean ERP waveforms of the auditory stimulus trials (red), visual stimulus trials (blue), and motor control condition (green), averaged across left temporal electrodes (FC3, FC5, FT7, C3, C5, T7, CP3, CP5, and TP7), frontocentral electrodes (F1, Fz, F2, FC1, FCz, FC2, C1, Cz, and C2), and right temporal electrodes (FC4, FC6, FT8, C4, C6, T8, CP4, CP6, and TP8). (B) Motor-subtracted difference waveforms of the auditory and visual stimulus trials, each plotted with 95% confidence intervals. Scalp topographies, derived from motor-subtracted difference waveforms of the auditory and visual stimulus trials, are overlaid on the waveforms.

ERPs for the centroparietal ROI

Figure S2 shows the ERP waveforms for the auditory and visual omission trials in the centroparietal ROI (C1, Cz, C2, CP1, CPz, CP2, P1, Pz, and P2). Similar to the results of the one-tailed one-sample *t*-tests against zero in the frontocentral ROI, the amplitude of the oP3 time window in the centroparietal ROI was not significantly different from baseline for either the auditory ($M = -0.19$ µV, $SD = 1.53$ µV) and the visual modality ($M = -0.41$ µV, $SD = 2.02$ µV), $t(32) = -0.73$, $p = 0.763$, $dz = -0.13$, $BF_{+0} = 0.12$; $t(32) = -1.17$, $p = 0.874$, $dz = -0.20$, $BF_{+0} = 0.09$, respectively.

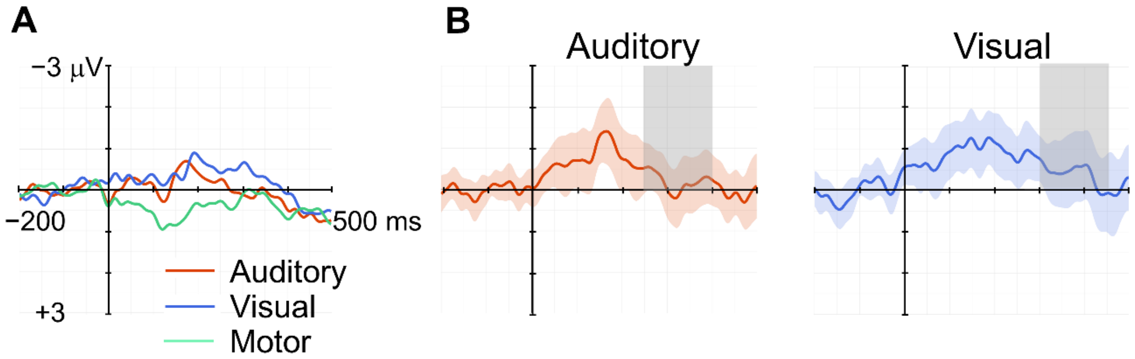

FIGURE S2. Grand mean ERP waveforms and difference waveforms (motor-corrected) of omission trials across centroparietal electrodes (C1, Cz, C2, CP1, CPz, CP2, P1, Pz, and P2). (A) Grand mean ERP waveforms of the auditory omission trials (red), visual omission trials (blue), and motor condition (green). (B) Motor-subtracted difference waveforms of the auditory and visual omission trials, each plotted with 95% confidence intervals.

Button press intervals for the first and second positions

Button press intervals and button press error rates were subjected to repeated measures analyses of variance (ANOVAs) with factors of sensory modality and sequence position. Here, the auditory modality refers to trials preceding auditory stimuli (including omissions), while the visual modality refers to trials preceding visual stimuli (including omissions). The first position refers to trials in which a modality was switched (A1 trials, V1 trials), and the second position refers to trials in which the same modality was repeated (A2 trials, V2 trials). For multiple comparisons, Bonferroni correction was applied to control for the type I error rate (α). Corresponding Bayesian repeated measures ANOVAs were conducted. Bayesian two-sided paired *t*-tests were performed when an interaction effect was significant on the ANOVAs. For the Bayesian repeated measures ANOVAs, the prior distribution for δ was modeled using a Cauchy distribution with a scale parameter of $r = 0.5$ for the fixed effects and $r = 1$ for random effects.

Table S1 shows the mean button press intervals and button press error rates in the first and second positions for the auditory and visual modalities. The button press intervals were significantly longer in the auditory modality than in the visual modality, and in the first than in the second position, $F(1, 32) = 5.19$, $p = 0.029$, $\eta_p^2 = 0.14$, $BF_{incl} = 29.40$, and $F(1, 32) = 97.90$, $p < 0.001$, $\eta_p^2 = 0.75$, $BF_{incl} > 100$, respectively. Additionally, the interaction effect was significant, $F(1, 32) = 7.18$, $p = 0.012$, $\eta_p^2 = 0.18$, $BF_{incl} > 100$. The post-hoc *t*-tests revealed that the mean button press intervals were significantly longer in the first position than in the second position for both the auditory, $t(32) = 7.03$, $p_{bonf} < 0.001$, $dz = 0.26$, $BF_{10} > 100$, and visual modalities, $t(32) = 3.06$, $p_{bonf} = 0.026$, $dz = 0.10$, $BF_{10} = 8.79$. Furthermore, in the first position, the mean button press intervals were significantly longer in the auditory than in the visual modality, $t(32) = 3.17$, $p_{bonf} = 0.020$, $dz = 0.12$, $BF_{10} = 11.08$, whereas in the second position, the difference was not significant , $t(32) = -1.35$, $p_{bonf} = 1.00$, $dz = -0.04$, $BF_{10} = 0.42$. For the mean button press error rates, neither the main effects nor the interaction effect were significant, $ps > 0.558$.

Table S1

Means and standard deviations of button press intervals and button press error rates in the first and second positions for auditory and visual trials

	Auditory		Visual	
	First position	Second position	First position	Second position
	M (SD)		*M* (SD)	
Button press interval (ms)	831 (116)	803 (111)	818 (112)	807 (111)
Button press error rate (%)	0.26 (0.39)	0.27 (0.39)	0.25 (0.38)	0.28 (0.41)

Note. The button press interval represents the time interval from the preceding button press, while the button press error indicates the proportion of trials in which the button press interval was shorter than 800 ms or longer than 2400 ms across all trials.

In both auditory and visual trials, the button press intervals were significantly longer in the first position (Auditory: 831 ms; Visual: 818 ms) than in the second position (Auditory: 803 ms; Visual: 807 ms). This suggests that participants unconsciously grouped button presses in pairs according to the sensory modality of the upcoming stimulus, as the overall difference was relatively small—only on the order of tens of milliseconds in behavioral measures. Furthermore, the button press intervals for the first position were significantly longer in auditory trials (831 ms) than in visual trials (818 ms). This result may reflect the fact that signals reach the sensory cortex more slowly for visual modality than for auditory modality. In other words, the time required to process the visual stimulus of preceding V2 trials before A1 trials may have been longer than the time required to process the auditory stimulus of preceding A2 trials before V1 trials, leading to a delayed button press in A1 trials compared to V1 trials as the action to generate the next stimulus. The difference in button press intervals between A1 and V1 trials was 13 ms, which is comparable to the modality differences in peak latencies between modalities (14.7 ms for oN1 and 10.4 ms for oN2). Additionally, although the difference was not statistically significant, the button press interval for V2 trials (807 ms) (preceded by V1 trials) was 4 ms longer than that for A2 trials (803 ms) (preceded by A1 trials), showing the same trend.

ERPs for the first and second positions

Figure S3 shows the motor-subtracted difference ERP waveforms for the auditory and visual omission trials and stimulus trials depicted separately for each sequence position (A1 trials vs. A2 trials, or V1 trials vs. V2 trials). The upper two panels show the waveforms of omission trials, and the lower two panels show the waveforms of stimulus trials. Two-tailed paired *t*-tests were conducted for the mean amplitudes of the difference waveforms to examine whether there were differences between omission trials in the first position (A1 trials, V1 trials) and in the second position (A2 trials, V2 trials) for the auditory and visual modalities separately. Table S2 shows the mean amplitudes and the results of these statistical analyses. The results indicate that neither the auditory nor the visual oN1, nor the auditory oN2 amplitudes differed significantly between the first and second positions. Only the amplitude of the visual oN2 differed significantly between the first and second positions, although the evidence was anecdotal.

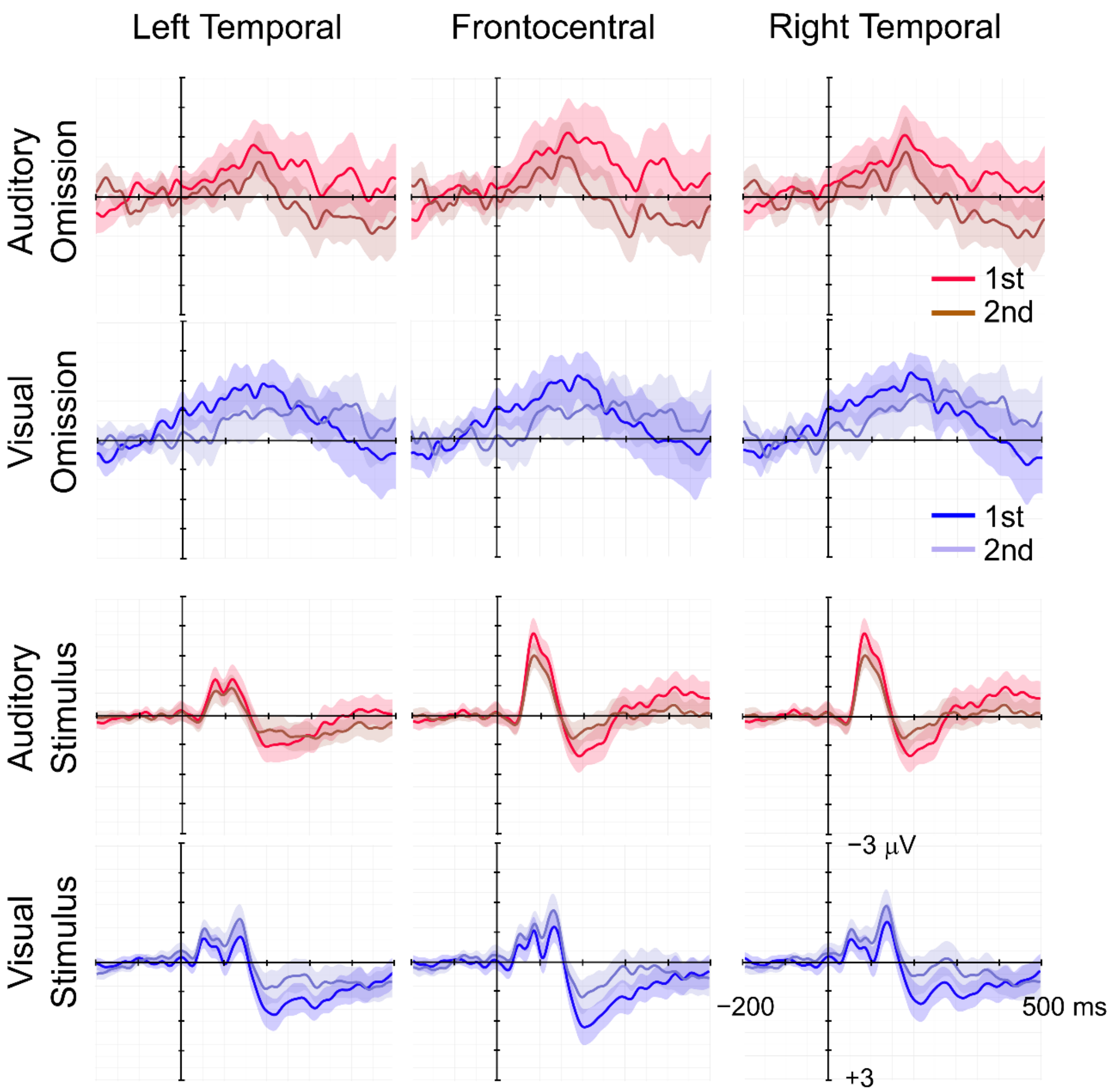

FIGURE S3. Motor-subtracted difference waveforms for omission trials (upper two panels: red for auditory, blue for visual) and stimulus trials (lower two panels: red for auditory, blue for visual) at the first and second positions, each plotted with 95% confidence intervals.

Table S2.

Comparison of Mean ERP Amplitudes between the first and second positions

$N = 33$	1st M (SD)	2nd M (SD)	t $df = 32$	p	dz	BF_{10}
Auditory oN1 (Frontocentral ROI)	−1.09 (2.10)	−0.65 (1.91)	−1.33	0.195	−0.23	0.41
Visual oN1 (Frontocentral ROI)	−0.76 (1.97)	−1.20 (1.71)	1.30	0.203	0.23	0.40
Auditory oN2 (Right temporal ROI)	−1.40 (2.07)	−0.86 (2.21)	−1.40	0.172	−0.24	0.45
Visual oN2 (Right temporal ROI)	−1.11 (1.85)	−1.65 (1.33)	2.28	0.030	0.40	1.75

Note. Results of paired t-tests and Bayesian paired t-tests are shown.

OSP peak latencies for the first and second positions

Figure S4 shows the results of peak latency comparisons for oN1 and oN2 between modalities depicted separately for each sequence position (A1 trials vs. V1 trials, or A2 trials vs. V2 trials). Modality × Position ANOVAs on the OSP peak latencies for oN1 and oN2 were performed. The oN1 peak latency was numerically shorter in the auditory modality (100.0 ms) than in the visual modality (107.2 ms), but none of the main effects of modality, position and the interaction were significant, *ps* > 0.106. The oN2 peak latency was significantly shorter in the auditory omission (192.5 ms) than in the visual omission trials (200.9 ms), $F(1, 32) = 6.50$, $p = 0.016$, $\eta_p^2 = 0.17$, $BF_{incl} = 1.51$. None of the main effects of position and the interaction were significant for oN2 latencies, *ps* > 0.307.

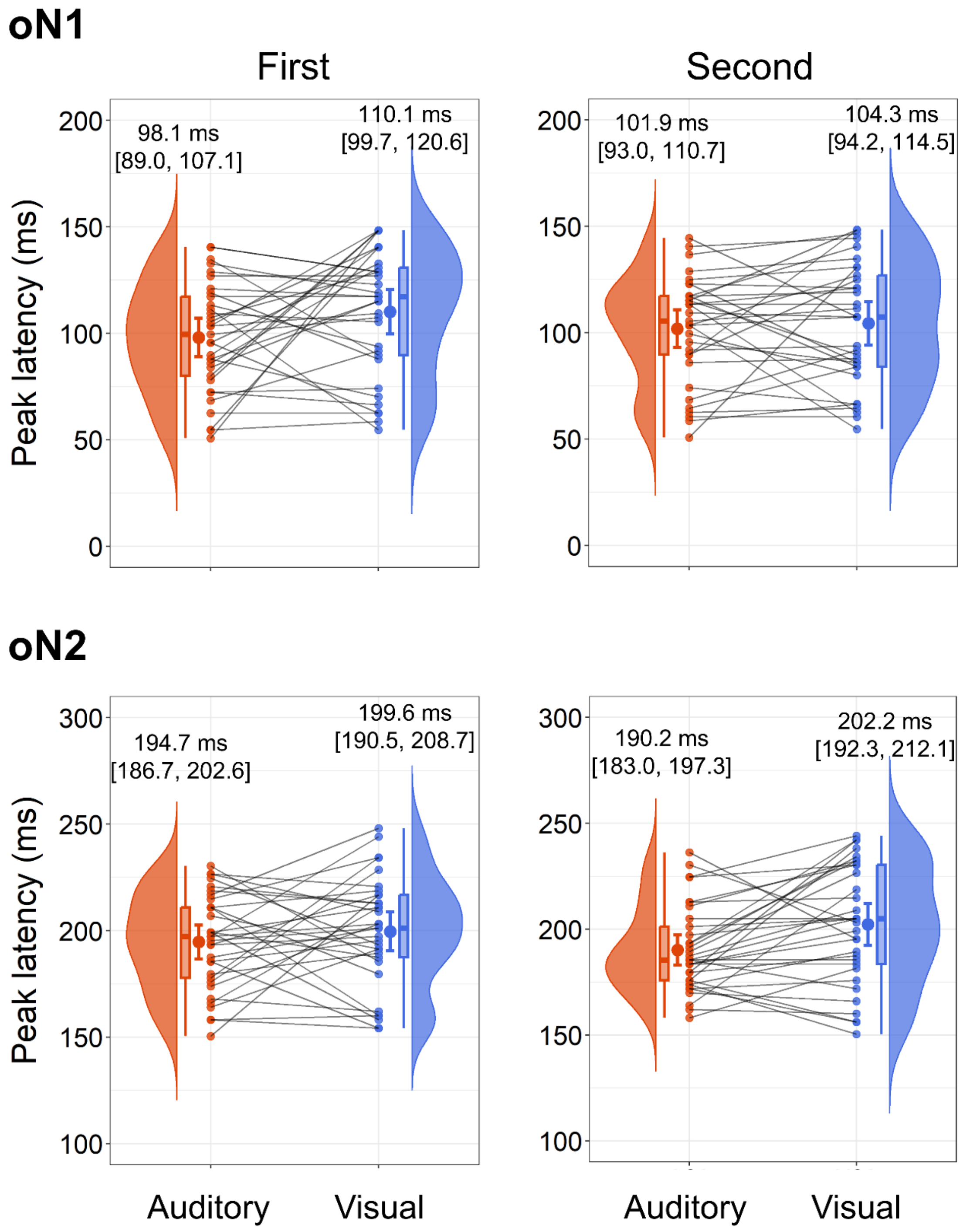

FIGURE S4. Comparisons of oN1 and oN2 peak latencies between the auditory and visual omission trials at the first and second positions. In both modalities, peak latencies were identified for each participant on the motor-corrected waveforms at T8, at each position, as the most negative peak within the time window of 50–150 ms for oN1, and 150–250 ms for oN2. Above the raincloud plots, the mean peak latencies with their 95% confidence intervals are shown.
